# Supplementary material for: Productive Performance and Cecum Microbiota Analysis of Broiler Chickens Supplemented with β-Mannanases and Bacteriophages—A Pilot Study
Source: Animals (Basel). 2022 Jan 11;12(2):169. doi: 10.3390/ani12020169 (PMC8772565; doi:10.3390/ani12020169)
Supplement: Supplementary file 1 [file animals-12-00169-s001.zip › animals-1481497-supplementary.pdf]

Table S1. Main bacterial abundance at Phylum level of the cecal content of broilers fed with CON, MN, BP, and MN + BP.

Phylum	Groups<br>CON	MN	BP	MN+BP	SEM	*P-value*
	Relative abundance %					
Euryarchaeota	0.03	0.47	1.24	0.02	0.30	0.85
Unassigned	0.04	0.03	0.11	0.04	0.02	0.88
Actinobacteria	7.74	8.15	2.49	7.29	2.06	0.94
Bacteroidetes	10.06	8.55	8.29	12.29	1.88	0.96
Cyanobacteria	1.73	0.75	1.15	0.90	0.26	0.86
Deferribacteres	0.13	0.06	0.00	0.09	0.04	0.80
Firmicutes	71.67	67.10	79.43	76.90	2.98	0.58
Proteobacteria	6.97	14.53	7.12	2.16	2.00	0.06
Tenericutes	1.62	0.34	0.18	0.30	0.32	0.79
Verrucomicrobia	0.01	0.01	0.00	0.00	0.00	0.59

CON: Corn-soy meal + 10 ppm antibiotic growth promoter (Enramycine, Enradin® F80), MN: as CON + 500 ppm β-mannanases, BP: corn-soy meal + 500 ppm of Bacteriophage Cocktail, MN + BP: as BP + 500 ppm β-mannanases + 500 ppm of Bacteriophage Cocktail. SEM: Standard Error of the Mean

Table S2. Main bacterial abundance at Family level of the cecal content of broilers fed with CON, MN, BP, and MN + BP.

Phylum	Family	Groups<br>CON	MN	BP	MN+BP	SEM	P-value
		Relative abundance %					
Euryarchaeota	Methanobacteriaceae	0.00	0.37	1.25	0.00	0.30	0.58
	Unassigned	0.04	0.03	0.11	0.04	0.02	0.88
Actinobacteria	Corynebacteriaceae	0.19	0.03	0.03	0.02	0.04	0.78
	Dermabacteraceae	0.26	0.03	0.01	0.01	0.06	0.40
	Bifidobacteriaceae	6.94	7.90	2.15	7.03	2.06	0.99
	Coriobacteriaceae	0.33	0.22	0.31	0.24	0.03	0.76
Bacteroidetes	Bacteroidaceae	0.38	0.28	0.96	2.36	0.40	0.82
	Porphyromonadaceae	0.05	0.50	0.95	0.01	0.19	0.90
	Rikenellaceae	8.48	6.92	5.33	9.57	1.55	0.90
	S24-7	0.58	0.17	0.54	0.15	0.13	0.87
	[Barnesiellaceae]	0.32	0.53	0.31	0.09	0.11	0.59
	[Odoribacteraceae]	0.28	0.19	0.23	0.13	0.04	0.86
Cyanobacteria	YS2;f_NA	1.74	0.75	1.16	0.90	0.26	0.86
Deferribacteres	Deferribacteraceae	0.13	0.06	0.00	0.09	0.04	0.80
Firmicutes	Bacillaceae	0.75	0.07	0.63	0.22	0.21	0.43
	Staphylococcaceae	0.26	0.09	0.05	0.07	0.04	0.38
	Enterococcaceae	0.08	0.09	0.14	0.05	0.03	0.80
	Lactobacillaceae	2.25	1.33	4.36	1.45	0.46	0.43
	Streptococcaceae	0.07	0.06	0.36	0.21	0.06	0.68
	Clostridiales;f_NA	7.32	4.54	5.43	4.85	0.45	0.33
	Christensenellaceae	0.09	0.07	0.11	0.13	0.02	0.93
	Clostridiaceae	0.04	0.05	0.11	0.09	0.01	0.33
	Dehalobacteriaceae	0.05	0.04	0.06	0.09	0.01	0.34
	Lachnospiraceae	21.34	18.20	23.62	20.47	1.65	0.73

	Peptostreptococcaceae	0.38	0.16	0.43	0.29	0.08	0.66
	Ruminococcaceae	34.33	31.08	37.89	40.76	3.16	0.73
	Veillonellaceae	3.53	10.55	5.38	7.28	2.12	0.80
	[Mogibacteriaceae]	0.19	0.12	0.12	0.14	0.02	0.36
	Erysipelotrichaceae	1.02	0.77	0.82	0.82	0.10	0.84
Proteobacteria	Alcaligenaceae	0.42	0.44	0.31	0.09	0.09	0.59
	Desulfovibrionaceae	0.34	0.20	0.30	0.10	0.05	0.30
	Campylobacteraceae	0.11	0.07	0.07	0.05	0.02	0.30
	Helicobacteraceae	2.60	3.97	1.74	0.62	0.79	0.79
	Enterobacteriaceae	3.49	9.77	4.60	1.28	1.91	0.40
Tenericutes	Anaeroplasmataceae	1.45	0.24	0.05	0.10	0.31	0.23
	RF39:f_NA	0.17	0.10	0.11	0.20	0.03	0.60

CON: Corn-soy meal + 10 ppm antibiotic growth promoter (Enramycine, Enradin® F80), MN: as CON + 500 ppm β-mannanases, BP: corn-soy meal + 500 ppm of Bacteriophage Cocktail, MN + BP: as BP + 500 ppm β-mannanases + 500 ppm of Bacteriophage Cocktail. SEM: Standard Error of the Mean

Table S3. Main bacterial abundance at Genus level of the cecal content of broilers fed with CON, MN, BP, and MN + BP.

Phylum	Genus	Groups					
		CON	MN	BP	MN + BP	SEM	P-value
		**Relative abundance %**					
Euryarchaeota	Methanobrevibacter	0.00	0.37	1.25	0.00	0.30	0.58
	Unassigned	0.04	0.03	0.11	0.04	0.02	0.88
Actinobacteria	Corynebacterium	0.19	0.03	0.03	0.02	0.04	0.77
	Brachybacterium	0.26	0.03	0.01	0.01	0.06	0.07
	Bifidobacterium	6.96	7.91	2.15	7.04	2.06	0.99
	Coriobacteriaceae;g_NA	0.25	0.18	0.27	0.21	0.03	0.64
Bacteroidetes	Bacteroides	0.38	0.28	0.96	2.36	0.40	0.82
	Parabacteroides	0.05	0.50	0.95	0.01	0.19	0.90
	Rikenellaceae;_NA	6.26	5.54	2.87	9.01	1.52	0.87
	AF12	0.37	0.44	0.18	0.03	0.08	0.45
	Alistipes	0.16	0.23	0.70	0.07	0.13	0.77
	Rikenella	1.70	0.72	1.60	0.46	0.40	0.55
	S24-7;g_NA	0.58	0.17	0.54	0.15	0.13	0.87
	[Barnesiellaceae];g_NA	0.33	0.53	0.31	0.09	0.11	0.59
	Odoribacter	0.28	0.19	0.23	0.13	0.04	0.86
Cyanobacteria	YS2;f_NA;g_NA	1.74	0.75	1.16	0.90	0.26	0.86
Deferribacteres	Mucispirillum	0.13	0.06	0.00	0.09	0.04	0.80
Firmicutes	Bacillaceae;g_NA	0.74	0.07	0.63	0.22	0.21	0.46
	Staphylococcus	0.25	0.09	0.05	0.07	0.04	0.33
	Enterococcus	0.08	0.09	0.14	0.05	0.03	0.87
	Lactobacillus	2.25	1.33	4.37	1.45	0.46	0.43
	Streptococcus	0.07	0.06	0.36	0.21	0.06	0.68
	Clostridiales;f_NA;g_NA	7.33	4.54	5.44	4.86	0.45	0.33
	Christensenellaceae;g_NA	0.09	0.07	0.11	0.13	0.02	0.93
	Clostridium	0.04	0.03	0.11	0.08	0.01	0.09
	Lachnospiraceae;g_NA	9.97	6.15	8.49	8.96	1.10	0.16
	Blautia	2.59	2.21	2.35	2.30	0.28	0.97
	Clostridium	0.35	0.42	0.48	0.76	0.10	0.73
	Coprococcus	2.27	2.17	3.34	1.50	0.36	0.56
	Dorea	1.75	1.13	1.21	0.98	0.20	0.23

	[Ruminococcus]	4.44	6.14	7.76	6.00	0.80	0.54
	Peptostreptococcaceae;g_NA	0.38	0.16	0.43	0.29	0.08	0.66
	Ruminococcaceae;g_NA	9.80	7.62	9.43	9.11	0.87	0.83
	Anaerofilum	0.13	0.06	0.08	0.11	0.03	0.88
	Anaerotruncus	0.31	0.17	0.17	0.12	0.06	0.82
	Butyricicoccus	1.28	0.91	3.85	0.95	0.59	0.60
	Faecalibacterium	11.14	12.31	11.42	22.32	2.29	0.58
	Oscillospira	7.40	5.22	7.94	4.27	0.57	0.06
	Ruminococcus	3.82	3.16	2.73	2.73	0.34	0.29
	Subdoligranulum	0.49	1.66	2.30	1.20	0.51	0.54
	Megamonas	2.55	10.28	5.09	7.16	2.06	0.89
	Phascolarctobacterium	0.98	0.29	0.30	0.14	0.18	0.63
	[Mogibacteriaceae];g_NA	0.19	0.12	0.12	0.14	0.02	0.36
	Clostridium	0.27	0.17	0.26	0.28	0.04	0.83
	Coprobacillus	0.26	0.16	0.15	0.16	0.04	0.71
	Erysipelotrichaceae;g_NA	0.50	0.43	0.41	0.38	0.06	0.62
Proteobacteria	Sutterella	0.42	0.44	0.31	0.09	0.09	0.65
	Desulfovibrionaceae;g_NA	0.08	0.07	0.10	0.01	0.02	0.10
	Bilophila	0.07	0.01	0.20	0.08	0.04	0.90
	Desulfovibrio	0.18	0.09	0.00	0.00	0.04	0.12
	Campylobacter	0.11	0.07	0.07	0.05	0.02	0.78
	Helicobacter	2.60	3.98	1.74	0.62	0.79	0.79
	Enterobacteriaceae;g_NA	3.50	9.79	4.61	1.29	1.92	0.40
Tenericutes	Anaeroplasma	1.45	0.24	0.05	0.10	0.31	0.23
	RF39;f_NA;g_NA	0.17	0.10	0.11	0.20	0.03	0.60
Verrucomicrobia	Akkermansia	0.01	0.01	0.00	0.00	0.00	0.59

CON: Corn-soy meal + 10 ppm antibiotic growth promoter (Enramycine, Enradin® F80), MN: as CON + 500 ppm β-mannanases, BP: corn-soy meal + 500 ppm of Bacteriophage Cocktail, MN + BP: as BP + 500 ppm β-mannanases + 500 ppm of Bacteriophage Cocktail. SEM: Standard Error of the Mean
